# Prognostic power assessment of clinical parameters to predict neoadjuvant response therapy in HER2‐positive breast cancer patients: A machine learning approach

**DOI:** 10.1002/cam4.6512

**Published:** 2023-10-31

**Authors:** Annarita Fanizzi, Agnese Latorre, Domenica Antonia Bavaro, Samantha Bove, Maria Colomba Comes, Erika Francesca Di Benedetto, Federico Fadda, Daniele La Forgia, Francesco Giotta, Gennaro Palmiotti, Nicole Petruzzellis, Lucia Rinaldi, Alessandro Rizzo, Vito Lorusso, Raffaella Massafra

**Affiliations:** ^1^ I.R.C.C.S. Istituto Tumori “Giovanni Paolo II” Bari Italy

**Keywords:** bioinformatics, breast cancer, neoadjuvant therapy, prognostic factor

## Abstract

**Background:**

About 15%–20% of breast cancer (BC) cases is classified as Human Epidermal growth factor Receptor type 2 (HER2) positive. The Neoadjuvant chemotherapy (NAC) was initially introduced for locally advanced and inflammatory BC patients to allow a less extensive surgical resection, whereas now it represents the current standard for early‐stage and operable BC. However, only 20%–40% of patients achieve pathologic complete response (pCR). According to the results of practice‐changing clinical trials, the addition of trastuzumab to NAC brings improvements to pCR, and recently, the use of pertuzumab plus trastuzumab has registered further statistically significant and clinically meaningful improvements in terms of pCR. The goal of our work is to propose a machine learning model to predict the pCR to NAC in HER2‐positive patients based on a subset of clinical features.

**Method:**

First, we evaluated the significant association of clinical features with pCR on the retrospectively collected data referred to 67 patients afferent to Istituto Tumori “Giovanni Paolo II.” Then, we performed a feature selection procedure to identify a subset of features to be used for training a machine learning‐based classification algorithm. As a result, pCR to NAC was associated with ER status, Pgr status, and HER2 score.

**Results:**

The machine learning model trained on a subgroup of essential features reached an AUC of 73.27% (72.44%–73.66%) and an accuracy of 71.67% (71.64%–73.13%). According to our results, the clinical features alone are not enough to define a support system useful for clinical pathway.

**Conclusion:**

Our results seem worthy of further investigation in large validation studies and this work could be the basis of future study that will also involve radiomics analysis of biomedical images.

## 1 INTRODUCTION

Breast cancer (BC) is one of the most common cancers in women worldwide. About 15%–20% of breast cancers is classified as human epidermal growth factor receptor type 2 (HER2) positive.^1^ HER2‐positive BC are more likely to grow and spread than to HER2‐negative breast cancers.^2^ Specific treatments have been developed for HER2‐positive patients, like the monoclonal antibodies. The first monoclonal antibody to be used was trastuzumab, that blocks the proliferation of tumor cells, and the advent of this agent has revolutionized the therapeutic scenario of HER2+ disease.

A series of landmark clinical trials have shown that the introduction of trastuzumab to NAC may bring notable improvements as the pathologic complete response (pCR).^3^ More recently, the introduction of pertuzumab has carried to further advances characterized by both statistically and clinically meaningful benefit in pCR when trastuzumab—pertuzumab was used in the neoadjuvant setting, as well as in the adjuvant one—even though more modest.^7^ Moreover, it has been found that the introduction of pertuzumab to neoadjuvant therapy leads to larger benefits than NAC without pertuzumab.^8^, ^9^, ^10^

NAC consists of using chemotherapy to reduce a tumor's size before the main treatment, and the use of NAC has been historically associated with several advantages such as tumor downstaging to limit the extent of local surgery.^4^ Though it was initially used in locally advanced and inflammatory BC to limit the resection, this therapeutic strategy is now commonly used for earlier‐stage and operable breast cancer.^5^ However, despite the advantages, only 20%–40% of patients achieve pCR following NAC.^6^, ^7^, ^8^, ^9^ Starting from these premises, the goal of this study was to propose a machine learning model able to predict the pCR to NAC in HER2‐positive patients exploiting a subset of significant clinical features. Firstly, we used statistical tests to evaluate the significance of variables on the retrospective collected data. Then, we performed a feature selection procedure to identify a subset of features to be udes for training a machine learning‐based classification algorithm. An automated decision support tool able to predict NAC response for HER2‐positive breast cancer patients is very important to identify patients eligible for innovative therapeutic options when it is available.

## 2 MATERIALS AND METHODS

### 2.1 Experimental data

All HER2‐positive patients who received neoadjuvant chemotherapy including trastuzumab monotherapy or trastuzumab plus pertuzumab in the period 2018–2021 and referred to Istituto Tumori “Giovanni Paolo II” in Bari (Italy) were involved. Written consent was not required from subjects, as it is a retrospective study and involves minimal risk. All data were fully anonymized before analysis. Patients with carcinoma in situ and/or metastasis ab initio were excluded from this study. All patients collected for this study underwent a chemotherapy regimen combined with homogenous anti‐HER2 therapy. Indeed, all patients were subjected to taxol. For each patient, we collected the following features: age, menopausal status (premenopause/postmenopause), clinical lymph nodal status (clinical LN status, Neg/Pos), tumor multiplicity (Neg/Pos), histological grade (G, Elston–Ellis scale: 1, 2, 3), histological subtype (ductal, other types), estrogen receptor (ER, Neg/Pos and percentage values) status, progesterone receptor (Pgr, Neg/Pos and percentage values) status, cellular marker for proliferation (ki67, Neg/Pos with cut‐off 20% and percentage values), human epidermal growth factor receptor‐2 score (HER2 score: 0, 1+, 2+, 3+), human epidermal growth factor receptor‐2 status (HER2 status, Neg/Pos), pertuzumab (NAC CT, No/Yes). Finally, we collected the NAC pathologic complete response defined as absent residual invasive carcinoma in the breast and lymph nodes axillary after surgery.

### 2.2 Statistical analyses

For each characteristic, we evaluated the pCR as the ratio of patients with pathologic complete response after NAC and the 95% confidence interval (CI). All pCRs were represented in a forest plot. The statistical tests used to study the significance of variables respect to pCR are Fisher's exact test for categorical variables. Particularly, Fisher's exact test^11^ determines if there is a statistically significant correlation between two categorical variables and considers as null hypothesis that the variables are independent while as alternative hypothesis that the two variables are not independent.

The results with the *p*‐value less than 0.05 were considered statistically significant.

### 2.3 Classification model

To predict NAC response, we used the Random Forest (RF) machine learning method. RF algorithm is a classification method that aggregates the prediction by averaging, after joining the randomized decision trees. We performed the typical use of RF involving 100 trees and 20 features, as designated in Breiman.^12^ In order to reduce the over‐fitting risk, we fixed 5 observations per tree leaf.^13^, ^14^

Other well‐known classifiers were considered, but they did not improve the performances. Interim results were not reported to not burden the discussion. Starting from 11 features, that is, age, menopausal status, clinical lymph node status, multiplicity, grading, ER, Pgr, ki67, HER2/neu, and pertuzumab CT, we performed a feature selection procedure by means of a RF algorithm, which provides the impurity measure computed with the Gini's diversity index obtained permuting out‐of‐bag observations among the trees.^15^, ^16^ Thus, we selected all the features having a Gini's diversity index above the average of all the features set. Besides, for estimating missing data, we used the MissForest data imputation algorithm,^17^ a machine learning technique based on Random Forest algorithm and having many advantages. As a matter of fact, it can be used with mixed data types, both categorical and numerical, and it does not need assumptions of relationships between features. Therefore, within a 10 ten‐fold cross‐validation rounds, we evaluated the classification performances in terms of AUC, accuracy, sensitivity, and specificity, finding the optimal threshold using Youden's index test on the ROC curves.^18^

All the analyses were performed by using the MATLAB R2022a (MathWorks, Inc., Natick, MA, USA) software.

## 3 RESULTS

### 3.1 Statistical analysis

Table 1 shows clinical and treatment characteristics of the patient's sample consisting of 67 patients, whose 46.27% achieved pathologic complete response (pCR) at the end of NAC. Patients' age range is between 34 and 78 years, with a median age of 51 years. Besides, 41.79% of patients were treated with trastuzumab and the remainder with pertuzumab.

**TABLE 1:** Sample dataset characteristics.

Characteristic	Distribution	Total (*N* = 67)
Patient age	Median (1st–3th quantile)	51.0 (44.5–61.0)
Menopausal status	Pre‐menopausal (abs. %)	23 (34.33)
	Post‐menopausal (abs. %)	36 (53.73)
	Nan (abs. %)	8 (11.94)
Clinical LN status	Negative (abs. %)	26 (38.81)
	Positive (abs. %)	39 (58.21)
	Nan (abs. %)	2 (2.99)
Tumor multiplicity	No (abs. %)	45 (67.16)
	Yes (abs. %)	16 (23.88)
	Nan (abs. %)	6 (8.96)
Grading	I (abs. %)	–
	II (abs. %)	13 (19.40)
	III (abs. %)	44 (65.67)
	Nan (abs. %)	10 (14.93)
Grading	Ductal (abs. %)	62 (92.54)
	Other (abs. %)	5 (7.46)
ER (%)	Median (1st–3th quantile)	60 (0–90.00)
Pgr (%)	Median (1st–3th quantile)	0 (0–30)
Ki67 (%)	Median (1st–3th quantile)	35.00 (25.25–50.00)
HER2 score	2+ (abs. %)	13 (19.40)
	3+ (abs. %)	54 (80.60)
Pertuzumab CT	Yes (abs. %)	39 (58.21)
	No (abs. %)	28 (41.79)
pCR	Yes (abs. %)	31 (42.27)
	No (abs. %)	36 (53.73)

*Note*: For categorical variables, absolute (abs.) and percentage values (%) are reported in brackets. For continuous values, the median value and interquartile range (1st–3rd quantile) are reported in brackets.

**FIGURE 1:**
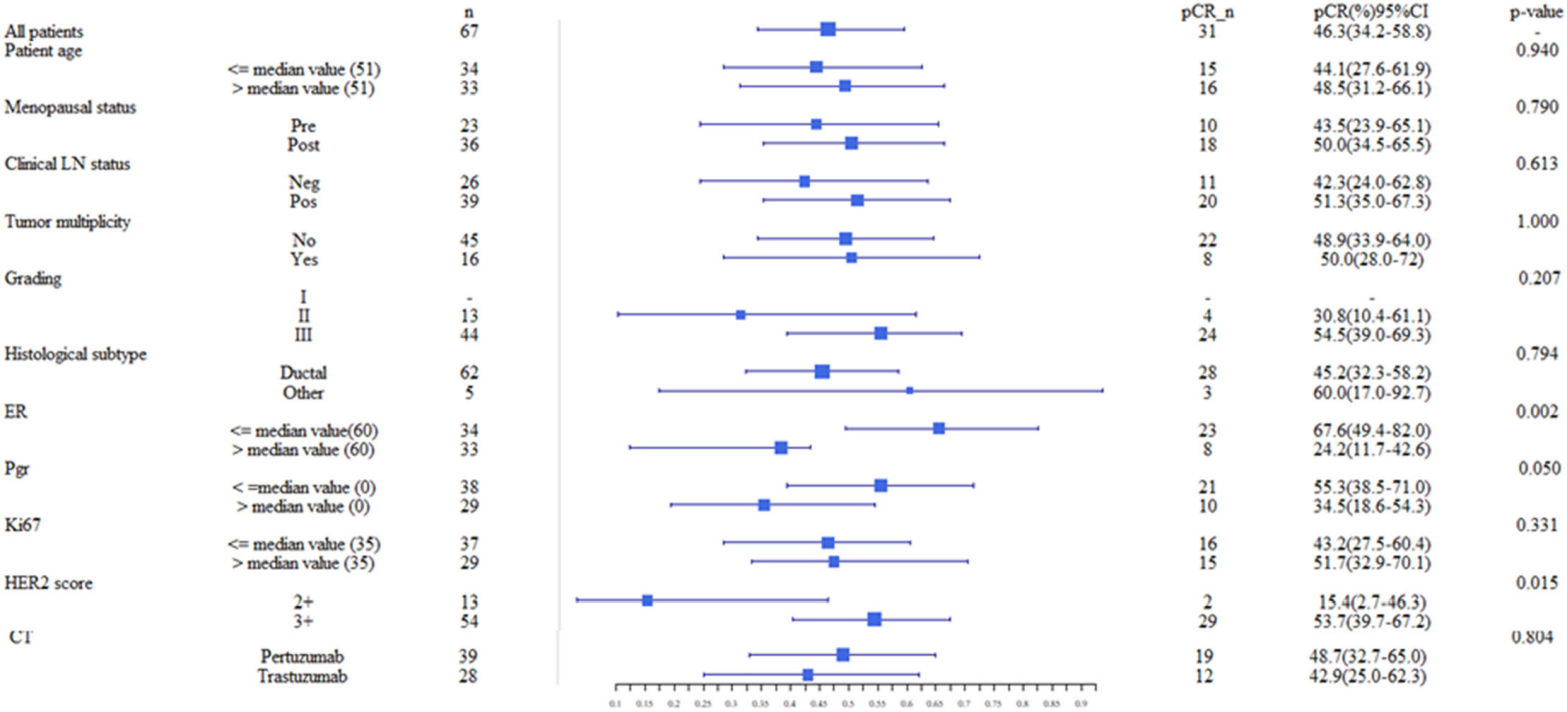
Forest plot of pathologic Complete Response (pCR). pCR and relative 95% Confidence Interval (CI) was calculated for several characteristics. The statistical tests used to study the significance of variables respect to pCR are Fisher's exact test for categorical variables. A result was considered significant when the *p*‐value was less than 0.05.

The only statistically significant variables with respect to the pCR were ER (*p*‐value of 0.002), Pgr (*p*‐value of 0.050), and HER2 score (*p*‐value of 0.050) (Figure 1). Particularly, patients with a tumor with high ER (%), Pgr positive and HER2 score of 3+ appear to respond worse to therapy. It should be noted that while PR median value was equal to 0, therefore corresponding to the cutoff conventionally used to discriminate the marker as positive or negative, the considered ER cutoff was equal to the median value (60%). Therefore, it emerges that in our real‐life sample, patients with ER much higher than the cut‐off conventionally used to binarize this biomarker, but below the median value of our sample, are more prone to respond to therapy. We also evaluated the pCR rate, considering the common positive and negative binarization for the ER. In this case, out of 47 patients with positive ER, only 18 patients responded (38.3%, 24.4–52.2 CI 95%), while out of 20 patients with negative ER, 13 patients responded (65.0%, 44.1–85.9 CI 95%), resulting statistically not associated with pCR (*p*‐value 0.062).

The other variables, including the type of NAC performed, did not show a statistically significant association with the pCR to neoadjuvant therapy.

### 3.2 Performance evaluation results

**FIGURE 2:**
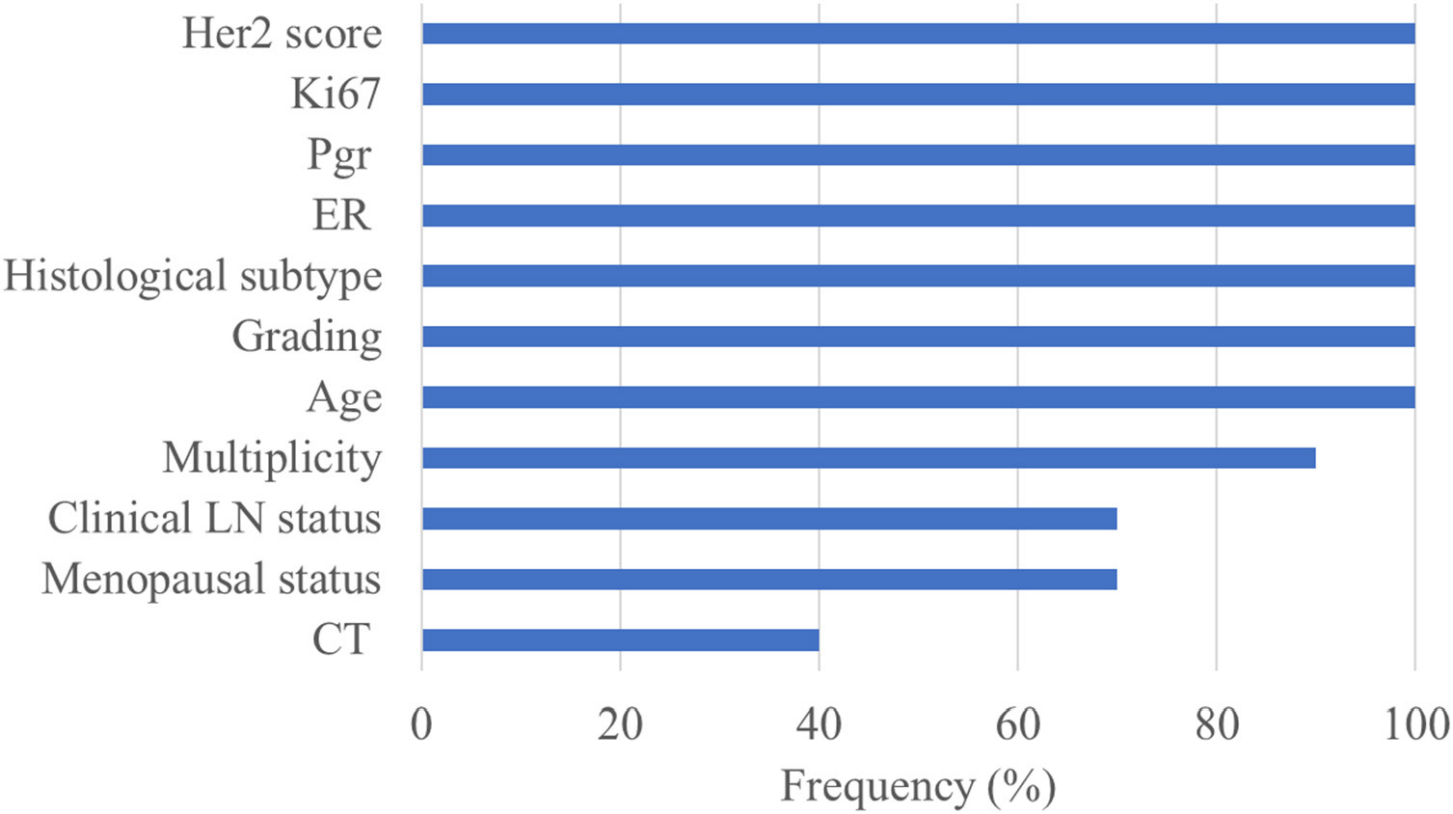
Distribution of selected features. Frequency distribution of the clinical features selected on 10 ten‐fold cross‐validation rounds by means of the RF feature selection algorithm.

Figure 2 shows the selection frequency of the clinical features within 10 ten‐fold cross‐validation rounds. Machine learning models, like all multivariate models, evaluate features in association with other variables. Therefore, features that when evaluated individually are not significantly associated with the outcome of interest, evaluated jointly with the others can increase their predictive value. Indeed, in addition to Er, PgR and HER2 scores that we had already verified as significantly associated with the pCR in the univariate analysis, also ki67, histological subtype, age at diagnosis and grading also assume an important discriminating power if evaluated jointly with the other characteristics. Multiplicity, clinical LN status, menopausal status, and NAC CT were the least frequently selected features. Additionally, in accordance with the previous statistical analysis, having or having not done pertuzumab appears not to be the least discriminating factor in predicting response to therapy.

**FIGURE 3:**
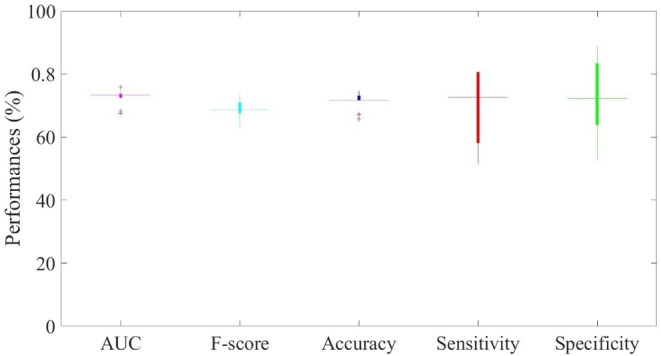
Classification performances. Distribution of AUC, accuracy, sensitivity, specificity, and F‐score was summarized. The results are obtained both through a 10 ten‐fold cross‐validation rounds.

Figure 3 represents the classification performances of RF classifier for the early prediction of NAC response in patients with HER2‐positive BC, based on the feature subset selected on each cross‐validation round. We summarized the performances metrics in terms of median value and 1st–3rd quartile. The proposed model reached an AUC of 73.27% (72.44%–73.66%) and an accuracy of 71.67% (71.64%–73.13%), with strong balancing between sensitivity and specificity, that is, 72.58% (58.06%–80.64%) and 72.22% (63.39%–83.33%), respectively.

## 4 DISCUSSION AND CONCLUSION

In the NAC setting, the use of trastuzumab‐based regimens for HER2‐positive BC led 39%–45% of patients to achieve pCR,^20^, ^21^ even if this percentage has been reported to widely vary between 25% and 65%, according to trials results previously mentioned.^22^ Pertuzumab has been recently approved for adjuvant therapy and NAC of early HER2‐positive BC.^7^, ^23^ Well‐known NEOSPHERE, KRISTINE, and PEONY clinical trials suggested that adding pertuzumab could improve pCR for HER2‐positive breast cancer.^8^, ^9^, ^10^

Several studies have been aimed at the validation of single biomarkers predicting the pCR in HER2‐positive BC patients.^22^, ^23^, ^24^, ^25^ In Ref.,^26^ authors identified factors associated with pCR in HER2‐positive breast cancer patients; they suggested that, even though both HER2 IHC and FISH are standard HER2 testing methods in BC, the success of pCR may be related with HER2 immunohistochemistry expression level.

In recent years, the development of artificial intelligence has led to important progresses in precision medicine, with these tools having the potential to aid clinicians obtain information on large and even very complex medical data—to make more accurate decisions and to finally improve clinical outcomes.^27^, ^28^ There are several artificial intelligence approaches proposed in the literature aimed at predicting the response to therapy, especially for breast‐related pathology. In our previous works, we developed predictive models aimed at predicting the response to neoadjuvant therapy in patients with breast cancer also using magnetic resonance images but without differentiating by cancer type.^29^, ^30^ Although the identification of a radiomic signature represents an added value in the definition of a personalized treatment plan, the prediction performances did not however exceed an AUC value of 78% on a private database of patients belonging to our Institute. The performances obtained in these previous works were comparable to those in the state of the art.^31^, ^32^ Nowadays, NAC is a standard therapy in clinical practice especially when the tumor is categorized as Triple‐Negative or HER2+.^33^, ^34^ In this prelaminar work, our goal was to evaluate clinical useful variables to direct therapeutic choices for a particular histological subtype of patients with HER2‐positive breast cancer. Our hypothesis is that differentiating the model performances by carcinoma type can be better. This is very important to select patients eligible for innovative therapeutic options when it is available. Indeed, a machine learning model able to better define predictors associated to the therapy response could influence the therapeutic choice for these patients in the next future.

Our experimental results show the pCR was significantly correlated with the ER, Pgr, and HER2 score. Especially, patients with higher ER, positive Pgr, and HER2 score equal to 3+ reported better responses. In our real‐life sample, the type of therapy, i.e., whether or not Pertuzumab was done, does not seem to be a factor associated with the response to NAC. The latter result contrasts with well‐known NEOSPHERE, KRISTINE, and PEONY clinical trials, but though it reflects the real‐life experience of our Institute, it could be due to the small number of the sample used.^8^, ^9^, ^10^

To evaluate the prediction power of the clinical features, we developed a machine learning model trained on a subgroup of significant features. The classification model reached a median AUC of 73.27% with a good balancing between sensitivity and specificity (72.58% and 72.22%, respectively). The features with higher prognostic power were those that identify the type of primary tumor. According to our results, the type of NAC (adding pertuzumab vs. only trastuzumab) does not seem to have an important informative power. Our real‐life results contrast with the results of large studies. However, it should be emphasized that the patients were recruited consecutively with the aim of defining a preliminary forecast project. Therefore, the lower pCR for patients receiving pertuzumab could be due to the composition of the study sample, relative to clinical parameters not considered in this study, such as lymph node status. Moreover, the clinical result could be biased by the small sample size. Nonetheless, in the proposed machine learning model, the variable referred to the type of chemotherapy performed (addition of Pertuzumab or not) is in any case a (binary) variable foreseen in the model definition phase which basically implicitly discriminates the cohorts of patients analyzed from the model. Therefore, in the future, the model suitably validated and optimized on larger cohorts will allow the re‐evaluation of this variable.

Although the model needs to be optimized in a larger database, the clinical features analyzed alone are not enough to define a tool useful for clinician in current clinical practice. Indeed, our study presents some limitations to be acknowledged. Among these, the sample size was relatively small, and the study lacked an independent validation cohort.

Nevertheless, we believe that the NAC response prognostic power of clinical features in Her2‐positive BC patients is not negligible.

Our results are encouraging and should be validated in a larger sample. In addition, by adding other clinical feature, for example, tumor stage and lymph node status, and the radiomic features we should achieve results with a higher performance to be able to define a tool, which could be applied to clinical practice.

This work represents the starting point of a future study that foresees the integration of quantitative characteristics extracted from pre‐treatment radiological images, in order to define a personalized medicine model. At the state of the art, the emerging scientific interest in radiomics has led to significant results in the field of early prediction of the response to NAC.^28^, ^29^, ^30^, ^31^, ^32^, ^33^, ^34^, ^35^, ^36^ In the feature work, we will apply our experience on radiomic analysis to HER2‐positive patients. Specifically, in order to improve the classification performance and therefore define a useful system for defining personalized therapeutic plans, we will integrate clinical features with radiomic ones.

## AUTHOR CONTRIBUTIONS

**Annarita Fanizzi:** Conceptualization (equal); data curation (equal); formal analysis (equal); methodology (equal); software (equal); validation (equal); writing – original draft (equal). **Agnese Latorre:** Data curation (equal); funding acquisition (equal); project administration (equal); resources (equal); supervision (equal); writing – original draft (equal). **Domenica Antonia Bavaro:** Conceptualization (equal); data curation (equal); formal analysis (equal); methodology (equal); validation (equal); writing – original draft (equal); writing – review and editing (equal). **Samantha Bove:** Writing – original draft (equal); writing – review and editing (equal). **Maria Colomba Comes:** Writing – original draft (equal); writing – review and editing (equal). **Erica Francesca Di Benedetto:** Writing – original draft (equal). **Federico Fadda:** Writing – review and editing (equal). **Daniele La Forgia:** Writing – review and editing (equal). **Francesco Giotta:** Data curation (equal); supervision (equal); writing – review and editing (equal). **Gennaro Palmiotti:** Writing – review and editing (equal). **Nicole Petruzzellis:** Data curation (equal); writing – review and editing (equal). **Lucia Rinaldi:** Data curation (equal); writing – review and editing (equal). **Alessandro Rizzo:** Writing – review and editing (equal). **Vito Lorusso:** Data curation (equal); supervision (equal); writing – review and editing (equal). **Raffaella Massafra:** Formal analysis (equal); funding acquisition (equal); project administration (equal); resources (equal); supervision (equal); writing – original draft (equal); writing – review and editing (equal).

## FUNDING INFORMATION

This work was supported by funding from Italian Ministry of Health, Ricerca Corrente 2023 deliberation n.187/2023.

## CONFLICT OF INTEREST STATEMENT

The authors declare no competing interests.

## ETHICS APPROVAL AND CONSENT TO PARTICIPATE

The study was conducted according to the guidelines of the Declaration of Helsinki and approved by the Scientific Board of Istituto Tumori “Giovanni Paolo II”—Bari, Italy. The number of the Protocol approved by the Ethic Commitee of Istituto Tumori “Giovanni Paolo II” (Bari, Italy) is 1168/CE. The authors affiliated to Istituto Tumori “Giovanni Paolo II”, IRCCS, Bari are responsible for the views expressed in this article, which do not necessarily represent the ones of the Institute.

## INFORMED CONSENT STATEMENT

Informed consent was obtained from all subjects involved in the study and/or their legal guardian(s).

## Data Availability

The raw data supporting the conclusions of this article will be made available, by the corresponding author, upon reasonable request.
